# Identification of Potential Antitubulin Agents with Anticancer Assets from a Series of Imidazo[1,2-*a*]quinoxaline Derivatives: In Silico and In Vitro Approaches

**DOI:** 10.3390/molecules28020802

**Published:** 2023-01-13

**Authors:** Kapil Kumar Goel, Afzal Hussain, Mohammad A. Altamimi, Satyendra Kumar Rajput, Prince Prashant Sharma, Rajeev Kharb, Wael A. Mahdi, Syed Sarim Imam, Sultan Alshehri, Osamah Abdulrahman Alnemer, Anu Chaudhary

**Affiliations:** 1 Amity Institute of Pharmacy, Amity University, Sector 125, Noida 201301, Uttar Pradesh, India; 2 Department of Pharmaceutical Sciences, Gurukul Kangri (Deemed to be University), Haridwar 249404, Uttrakhand, India; 3 Department of Pharmaceutics, College of Pharmacy, King Saud University, Riyadh 11451, Saudi Arabia; 4 Department of Pharmaceutical Chemistry, Bhagwati College of Pharmacy, Baraut 250611, Uttar Pradesh, India

**Keywords:** anticancer, tubulin, azaheterocycles, molecular docking, molecular mechanics, virtual screening, in vitro screening

## Abstract

Computer-aided drug design is a powerful and promising tool for drug design and development, with a reduced cost and time. In the current study, we rationally selected a library of 34 fused imidazo[1,2-*a*]quinoxaline derivatives and performed virtual screening, molecular docking, and molecular mechanics for a lead identification against tubulin as an anticancer molecule. The computational analysis and pharmacophoric features were represented as **1A2**; this was a potential lead against tubulin, with a maximized affinity and binding score at the colchicine-binding site of tubulin. The efficiency of this lead molecule was further identified using an in vitro assay on a tubulin enzyme and the anticancer potential was established using an MTT assay. Compound **1A2** (IC_50_ = 4.33–6.11 µM against MCF-7, MDA-MB-231, HCT-116, and A549 cell lines) displayed encouraging results similar to the standard drug colchicine in these in vitro studies, which further confirmed the effectiveness of CADD in new drug developments. Thus, we successfully applied the utility of in silico techniques to identify the best plausible leads from the fused azaheterocycles.

## 1. Introduction

Tubulin is a highly overexpressed protein in almost all types of cancer and it is a validated anticancer drug target. Tubulin is composed of microtubules that exist as a heterodimer of α,β-tubulin [1]. These tubulin polymers exist in a dynamic equilibrium via polymerization and depolymerization. Thus, it provides a structural framework during the karyokinesis process of cellular division [2]. Despite cell division, tubulin plays a vital role in intracellular trafficking, cell migration, and angiogenesis. Tubulin also promotes cancer cell growth and prognosis [3]. Metabolically active cancer cells often evade programmed cell death and continue their growth and prognosis. In this context, drugs targeting tubulin or microtubules disrupt the tubulin–microtubule equilibrium and these allow apoptosis. To date, numerous approved or clinical candidates as tubulin inhibitors have been reported from both natural and synthetic origins that target the four major binding sites in tubulin protein, including the laulimalide, taxane, vinca alkaloid, and colchicine sites [4]. Among these, the colchicine-binding site (CBS) is one of the significant and most crucial pockets explored for developing potential tubulin polymerization destabilizers. The inhibitors exert their mechanism by disrupting the tubulin assembly and inhibiting microtubule formation. Two blockbuster drugs that act at the CBS are colchicine and combretastatin. These drugs inhibit cell division at the G2/M phase, disrupt the cell cycle, and allow cell death through necrosis or apoptosis [2,5].

To date, numerous heterocyclics have been explored in the development of CBS binding agents and these have been successfully screened for their anticancer potential. However, the clinical application of these agents has not yet been established [6].

Numerous heterocyclic compounds are documented in the literature that show significant anticancer activities [7]. Fused imidazoquinoxaline-based moieties are promising in several potential natural and synthesized anticancer compounds, and have been tested against a broad spectrum of cancer cell lines. Most synthesized imidazoquinoxaline-based anticancer compounds have shown an anticancer activity towards diverse cancer cell lines. However, the in vitro activity of these compounds has been reported to be suboptimal [8,9,10,11]. Thus, despite the great anticancer potential, the clinical implementation of these compounds is still lacking due to safety concerns and expected outcomes in clinical studies. Imidazoquinoxaline derivatives are reported to target various cellular functions in cell division such as tubulin polymerization, lymphocyte-specific protein tyrosine kinase, tyrosine kinase, and EGFR (epidermal growth factor receptor). Imiquimod (Aldara) was the first drug of the fused imidazoquinoxaline family to be approved by the USFDA (United States Food and Drug Administration) in 1997 for various types of cancer. This pharmacophore was utilized to develop EAPB203 and EAPB503 compounds to target tubulin polymerization. The drug is still under clinical investigations for other findings [11]. As a synthon, imidazoquinoxaline presents many possibilities for structural modifications and has received huge attention from many researchers for the development of new anticancer molecules with improved target specificity and efficacy.

**Figure 1:**
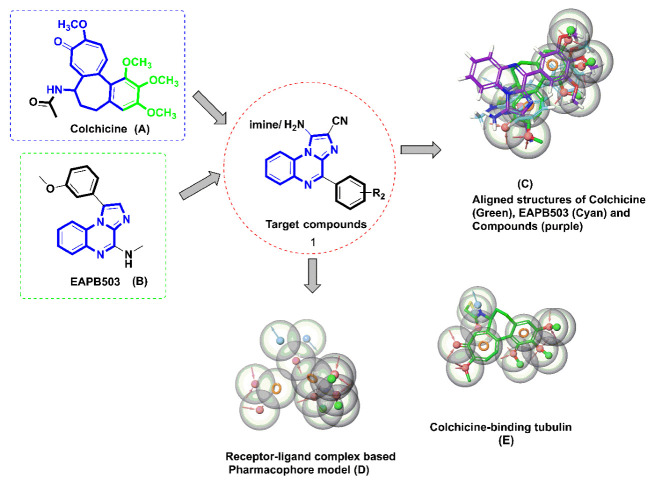
The rationale for selecting imidazo[1,2-*a*]quinoxaline pharmacophore as an anticancer agent via tubulin inhibition. This highlights the basis of selection of target compound (1) considering the pharmacophoric feature of approved tubulin inhibitor Colchicine (**A**) and investigational tubulin inhibitor, EAPB503 (**B**). The alignment of all the pharmacophore from (1, **A**,**B**) is represented (**C**) that reflects the aligned structures of Colchicine, EAPB503 and target compound. The critical overlapping was then refined that led to the generation of “Receptor-ligand complex” (**D**) using phase module. The obtained aligned pharmacophore was found to possess a hydrogen bond donor; a hydrogen bond acceptor; a hydrophobic group; an aromatic ring; a positively charged group; and a negatively charged group which is optimal for Colchicine binding at the CBS (**E**).

In the present work, we explored the library of imidazo[1,2-*a*]quinoxaline derivatives for their potential as tubulin polymerization destabilizers via in silico and in vitro approaches [12]. We rationally selected (Figure 1) imidazo[1,2-*a*]quinoxaline derivatives (1) considering the pharmacophoric features developed using a structure-based pharmacophore model. We developed a structure-based pharmacophore from the receptor-ligand complex option (e-pharmacophore) of a phase module with “PDB: 1SA0”. The pharmacophore was found to possess seven pharmacophoric points comprising: (a) one hydrogen bond donor (HBD) interacting with Thrα177; (b) three hydrogen bond acceptors (HBAs) wherein one interacted with Valα179, the second interacted with Cysβ239, and the third interacted with either or all, including Alaβ248, Aspβ249, and Leuβ250; (c) two hydrophobic centers; and (d) one planer region. For an optimal tubulin inhibitory activity, an identified pharmacophore must possess at least one hydrogen bond acceptor, a planer group, and two hydrophobic centers [6,13].

After the development of the pharmacophore, we screened the molecules of interest from the ligand database screening option of the phase module. All 34 selected imidazo[1,2-*a*]quinoxaline derivatives—which had substitutions with the phenyl ring at the −4 position, amino or iminic at the −1 position, and cyano at the −2 position as well as EAPB503 (investigational drugs)—possessed the essential features. All synthesized compounds were also found to have good phase fitness scores in the range of 2.7 to 2 in the pharmacophore-based screening. The phase fitness score measured how well the ligands matched the features of the developed pharmacophore, based on an alignment score, a vector score, and a volume score.

The pharmacophores and their binding model could provide critical insights for a rational structure-based drug design. Therefore, we developed a pharmacophore for one of our prototype’s compounds. The model revealed that the pharmacophore of our synthetic compound possessed six pharmacophore groups: a hydrogen bond donor (D); a hydrogen bond acceptor (A); a hydrophobic group (H); an aromatic ring (R); a positively charged group (P); and a negatively charged group (N). Thus, these satisfied our quest for an optimal pharmacophoric requirement among a series of developed imidazo[1,2-*a*]quinoxaline derivatives [14].

In the current work, we identified a potential putative tubulin inhibitor with an anticancer potential from a library of 34 fused imidazo[1,2-*a*]quinoxaline derivatives using computational methods (including virtual screening, molecular docking, and molecular mechanics). The molecules were rationally chosen considering the pharmacophoric features of colchicine and EAPB203—approved and investigational drugs, respectively—known to inhibit tubulin and possessing a potential anticancer activity. The identified compound with the best affinity was further validated using an in vitro-based biological technique as well as establishing the tubulin inhibitory and anticancer potentials (assets).

## 2. Results and Discussion

**Figure 2:**
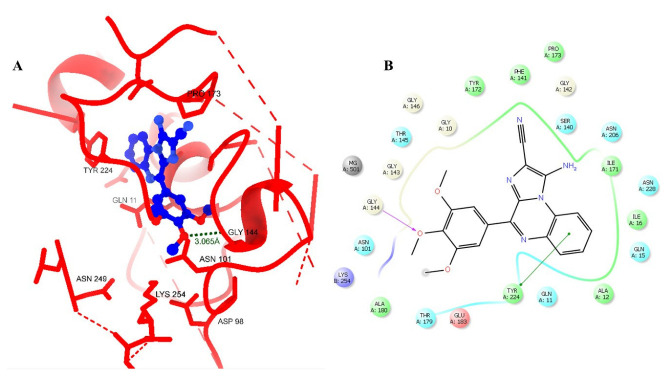
Illustration portraying the interaction pattern of **1A2**. (**A**) A 3D surface model and (**B**) a 2D interaction diagram within the colchicine-binding domain of tubulin (PDB: 1SA0).

The virtual screening-based molecular docking studies (Table 1) revealed that **1A2** exhibited a profound affinity within the colchicine-binding domain of the tubulin protein. The glide score for **1A2** was found to be −11.45 kcal/mol compared with colchicine, which portrayed a docking score of 9.15 kcal/mol. The binding and interaction pattern of **1A2** (Figure 2a,b) within the colchicine-binding site revealed fundamental interactions with CYS241, LEU248, LEU255, VAL318, ALA316, LYS352, MET259, and LYS254.

Furthermore, the re-analysis of the 34 ligands (Table 1) based on the fused imidazo[1,2-a]quinoxaline derivatives revealed that compounds **1B2** (−9.132 kcal/mol), **1C2** (−9.346 kcal/mol), **1C7** (−9.342 kcal/mol), and **1D2** (−9.168 kcal/mol) portrayed similar docking scores in comparison with colchicine (−9.156 kcal/mol), which was employed as the positive control among the aldehyde derivatives (**1A1**–**1D2**). A thorough analysis revealed that the presence of the electron-withdrawing group methoxy –OCH_3_), particularly at R_2_ and R_3_ (**1B2** and **1C2**), was more tolerable to execute a better affinity towards the colchicine-binding site. Other electron-withdrawing groups at the R_3_ positions (-NO_2_ in **1C7** and -CN in **1D2**) were also equally tolerable. These functional groups were found to interact with the CYS241, LEU248, LEU255, and VAL318 amino acids, which are critical in conformational stability within the active domain.

Compounds **1E3** (−9.486 kcal/mol), **1E7** (−9.358 kcal/mol), 1E8 (−9.546 kcal/mol), **1E12** (−9.432 kcal/mol), and **1E13** (−9.242 kcal/mol) were found to possess a comparable or better affinity concerning colchicine (−9.156 kcal/mol) within the colchicine-binding site of the tubulin, as deduced by the glide score among the ketone derivatives (**1E1**–**1E16**). The analysis suggested that the bulky group (R = phenyl) (**1E3**) was found to be tolerable in eliciting an affinity. Furthermore, the presence of –OCH3 substituents in the phenyl at R’ (**1E12** and **1E13**) was found to be tolerable in good binding with the receptor. The critical interactions of these compounds were further found to be in line with the active aldehydic derivative interactions of the amino acids (CYS241, LEU248, LEU255, and VAL318).

Molecular docking is concerned with the relative binding energies of each molecule, which simplifies the calculations by employing numerous approximations to achieve high and quick throughput screening. However, docking usually sacrifices accuracy in a quest to achieve a more incredible speed, thus necessitating proper rescoring steps to predict binding energies [15,16,17]. To predict the binding interactions of the selected ligands within the colchicine-binding site of the tubulin protein, we employed MMGBSA-based calculations. This method is accurate as it is based on a force field that calculates the free energy of binding rather than free energy perturbation (FEP) or thermodynamic integration, as employed by other computational techniques. Thus, the method yields better and more accurate results than docking. Subsequently, the current study also established the MMGBSA-binding energy of all the proposed molecules. Table 1 represents the MMGBSA-binding energy results as obtained from the current study. As evident from the results, we found a strong correlation for the best-docked lead **1A2** and **1E8**. Compounds **1D2**, **1E3**, **1E7**, **1E12**, and **1E13** possessed excellent dock scores. However, these failed to score well in the MMGBSA-binding energy calculations. Thus, the analysis put forth the need for better scoring calculations in addition to molecular docking to quantify the binding affinity with the binding energy.

**Figure 3:**
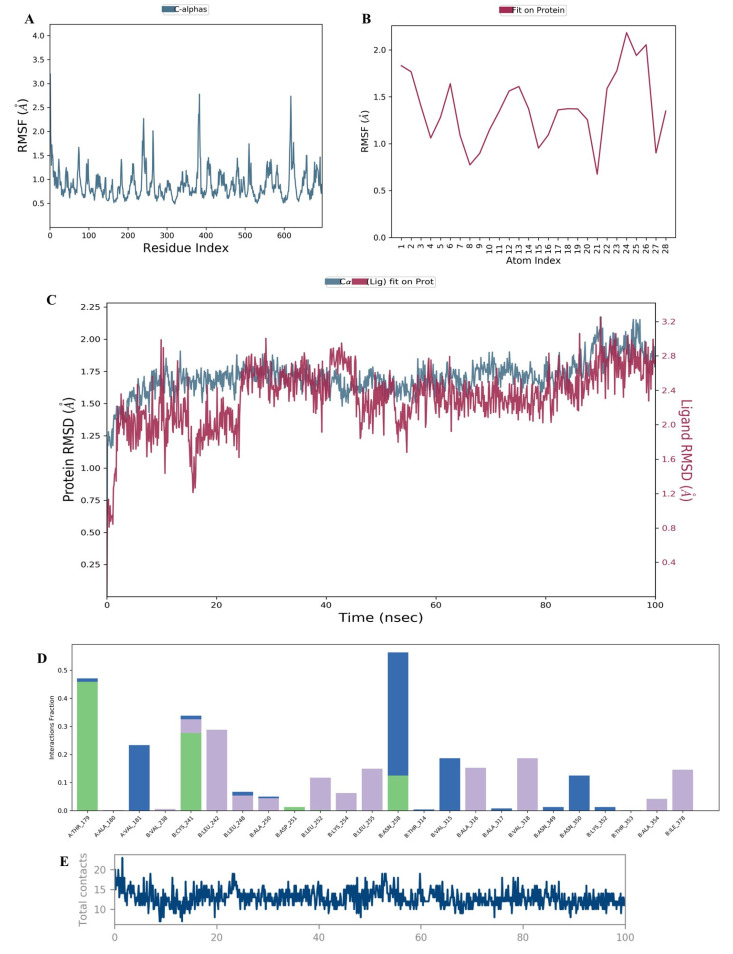
The outcomes of the MD analysis of ligand **1A2** with colchicine-binding domain of tubulin (PDB: 1SA0) over simulation period of 100 ns, represented by: (**A**) protein-based RMSF pattern; (**B**) ligand-based RMSF analysis; (**C**) perturbations recorded with ligand and its binding within CBS as indicated by change in RMSD; (**D**) protein–ligand contact diagram generated from hydrogen bonding, hydrophobic interactions, and formation of water bridge over the simulation period; (**E**) protein–ligand contact trajectory over the simulation time.

Ligands and receptors are in a few perturbations (such as in biological systems); thus, we analyzed whether **1A2** could retain a conformational stability via molecular dynamic (MD) studies or not. The simulations were performed and the trajectory of stability between the ligand–protein complexes was monitored for an extended duration of 100 ns. The findings are illustrated in Figure 3. To understand the protein chain perturbations and their impact on the protein–ligand stability, we calculated the RMSF (root mean square fluctuation) that determined the changes occurring in the protein residues throughout the simulation. The protein-based RMSF revealed (Figure 3A) amino acids in the range of 250–280, 380–400, and 600–630. These were slightly perturbed by 1–2.5 Å. The remaining amino acids were found to be stabilized with minimal fluctuations. Moreover, we analyzed the ligand RMSF (Figure 3B) that portrayed the changes occurring in the ligand atom positions due to the conformational changes, especially those concerned with its binding to the receptor. The analysis revealed fluctuations within a range of 1–2 Å, particularly due to the C-C rotations in the lead compound **1A2**. We then interpreted the RMSD-based MD analysis (Figure 3C), which monitored the average changes in the displacement of the ligand atoms with respect to the protein-binding site over a particular time frame. A time frame of 100 ns revealed that the RMSD value for the protein was 1.5 Å whereas it was 3.2 Å for the ligand at the end of the MD simulation. The RMSD fluctuation was 0.6 viz within an acceptable value of 2 Å. However, an initial drift was observed in the protein–ligand complex for a time duration of 27 ns. From the ligand–RMSF plot, it was understood that both the -CN groups of the ligand underwent maximum fluctuations during the MD run and they showed relatively poor interactions with the protein backbone compared to the rest of the ligand structure.

**Figure 4:**
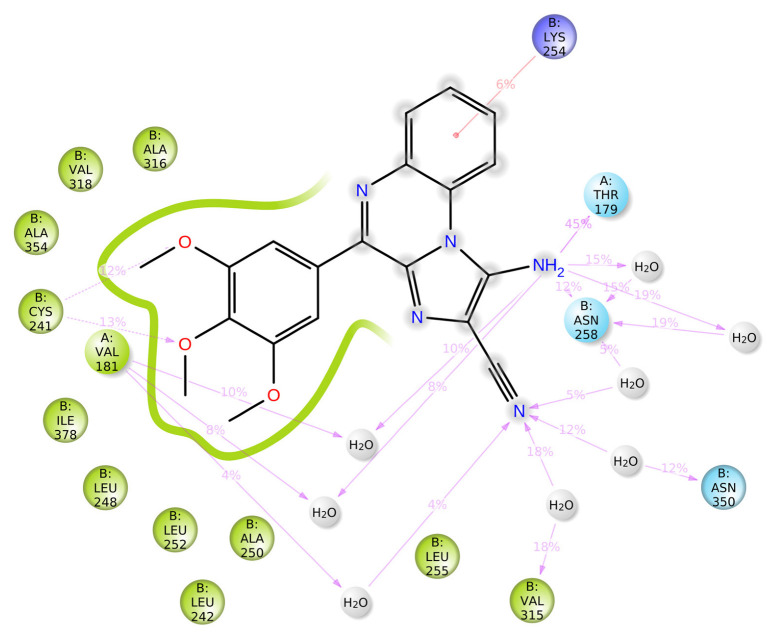
Time frame analysis of **1A2** with colchicine-binding domain of tubulin (PDB: 1SA0) over a simulation period of 30 ns.

From the protein–ligand (PL) contact diagram, the protein interaction with the ligands was tracked during a simulation period of 100 ns. The PL interaction diagram was divided into four interactions: hydrogen bonds, ionic bond, hydrophobic interaction, and water bridges. The PL contact diagram (Figure 3D) on the y-axis showed the interaction fraction. The x-axis showed the amino acid participating in whatever sort of interaction wherein the value of 0.7 showed 70% of the interaction maintained over the entire simulation. From the diagram (Figure 3D), we observed THR179, CYS241, ALA250, and ASN248 to be involved in H-bonding during the simulation time, wherein THR179 exhibited the highest interaction fraction with the **1A2** compound—˃70% over the course of 100 ns. The amino group of **1A2** took part in H-bonding with THR179. The amino acids participating in the hydrophobic interactions were LEU242, LEU252, LYS254, ALA316, VAL318, ALA354, and ILE378. The compound **1A2** interacted with LEU242 at around 80% during course of the simulation, which involved a cation-pi type of interaction with the aromatic ring of the ligand. Furthermore, we found that a stable trajectory (Figure 3E) was made by the interactions of the ligand with the protein. The P–L interaction diagram with a specific time frame analysis is represented in Figure 4.

**Figure 5:**
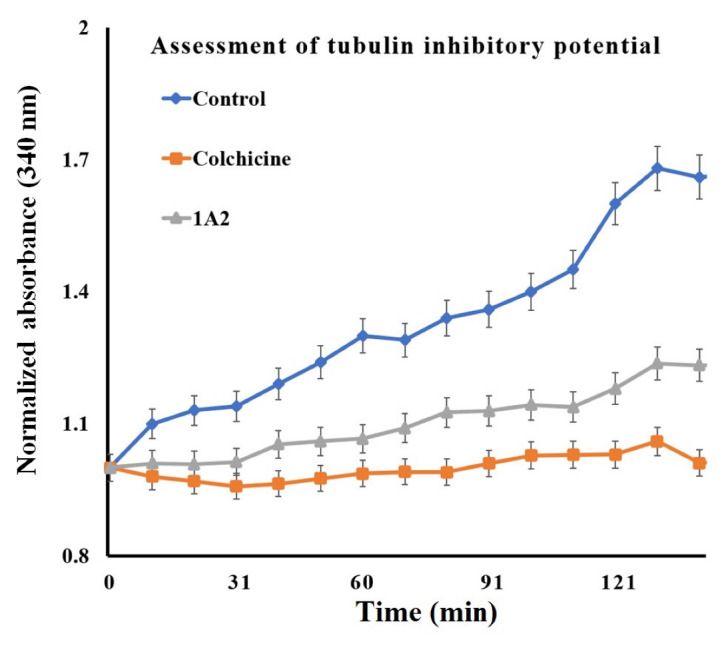
Graph suggesting the tubulin inhibitory potential of **1A2** compared with colchicine employed as a positive control for tubulin assay.

After obtaining a convincing outcome of an in silico interaction of **1A2** with CBS, we confirmed these outcomes using a biological (in vitro) technique. For this purpose, we chose our best ligand, **1A2,** obtained from the in silico-based results. The lead compound **1A2** was further subjected to a tubulin polymerization assay to determine its potential as a tubulin polymerization destabilizer. The analysis revealed (Figure 5) that compound **1A2** possessed similar tubulin polymerization inhibition properties to colchicine when compared with the control group. It was found to destabilize the polymerization of tubulin units similar to colchicine, but to a lesser extent. The results further encouraged the investigation of this compound as an anticancer agent in biological in vitro cell lines.

Based on the in silico outcomes, it was imperative to assess the tubulin inhibition potential of **1A2** for its anticancer potential. Therefore, we performed an MTT-based antiproliferative activity assay. We employed the MCF-7, MDA-MB-231, HCT-116, and A549 cell lines for our assay. The result of the MTT assay (Table 2) supported the in silico findings due to the cytotoxic character of this compound and it was presented as a potential pharmacophore to control cancer. Compound **1A2** showed a potential cytotoxicity against the MCF-7, MDA-MB-231, HCT-116, and A549 cell lines, with IC_50_ values of 4.33 ± 0.31 µM, 6.11 ± 0.23 µM, 5.87 ± 0.31 µM, and 5.44 ± 0.18 µM, respectively. The results were comparable with colchicine, which confirmed the anticancer potential of **1A2**.

## 3. Materials and Methods

### 3.1. Materials

The required chemicals and solvents (analytical grade) were obtained from Sigma Aldrich, Mumbai, and Maharashtra, India. Colchicine was procured from CDH Pvt. Ltd., New Delhi, India. The identified lead(s) were synthesized as per the protocol employed by Joshi et al. [12].

### 3.2. Methods

#### 3.2.1. Compound Selection and Virtual Screening

**Figure 6:**
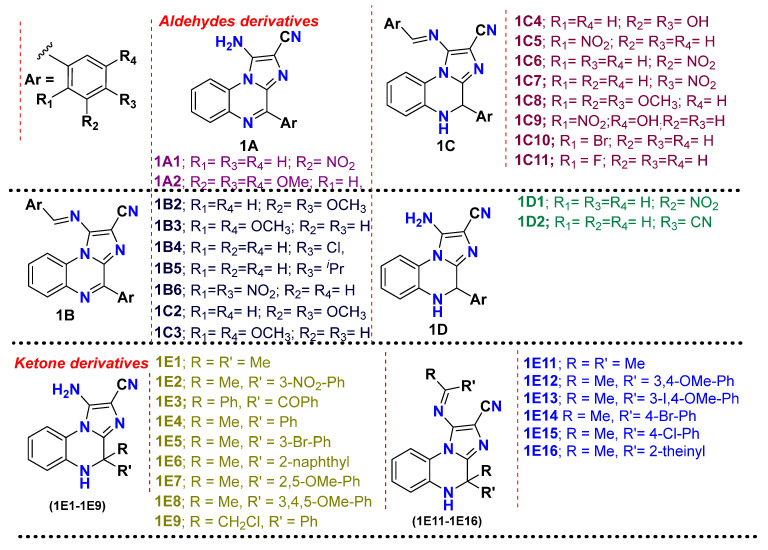
Selected chemical structures (34) belonging to the imidazo[1,2-*a*]quinoxaline category for the present work.

A total of 34 compounds belonging to imidazo[1,2-*a*]quinoxaline derivatives from a library [12] were selected for a virtual screening and molecular docking against the tubulin protein (PDB: 1SA0). The details of the chemical structure are provided in Figure 6. These derivatives (34) were further chosen for in silico-based studies to identify the best putative lead compound among them. Colchicine served as a positive control during the conducted studies.

At the initial stage of the drug development process, virtual screening is an important starting point of the lead identification process where molecular docking simulation studies are the most widely used. The various fundamental steps include the preparation of the protein, the validation of the protein, preparing the ligand molecules, receptor grid generation, and molecular docking simulation studies.

#### 3.2.2. Protein Preparation and Validation

The protein X-ray crystal structure of tubulin (PDB: 1SA0) was obtained from the protein data bank [18]. The protein was prepared using the protein preparation wizard of Schrodinger software (2021-2). This included the removal of water molecules, the addition of missing side chains and loops, optimization, the addition of hydrogen atoms, the generation of a proper order to the metal-based bonds, and a selenomethionine to methionine conversion. Finally, we initially optimized the protein and subsequently minimized it by OPLS_2005 as a force field whilst keeping the coverage heavy atoms to the RMSD (root mean square deviation) under the refining tab at 0.30 Å [19]. This process resulted in a protein structure with a minimum energy conformation.

#### 3.2.3. Grid Generation

The grid is the target volume of the protein where the docking will take place. After the protein preparation, the minimum protein was subjected to a grid generation using the receptor grid generation tool of Schrodinger Maestro. The co-crystallized ligand of the protein was used to set the grid dimensions. All atoms within 5 Å with a co-crystallized ligand in the crystal coordinates of tubulin were chosen as a binding site. Herein, a scaling factor of 1 and a partial cut-off charge of 0.25 were set. The remainder were kept under default conditions for the receptor grid generation.

#### 3.2.4. Ligand Preparation

The 2D structures of the ligands were drawn using ChemDraw Ultra (version 7.0.4, ChemOffice, Cambridge, MA, USA). The 3D structures of the ligands were prepared using the LigPrep wizard application of Schrodinger Maestro. Briefly, the initial 2D structures of the ligands were imported into the working window of Schrodinger. The Epik tool of LigPrep was used to generate the possible states at the target pH (7.4), followed by the tautomer generation. The OPLS_2005 force field was applied to provide the minimum energy conformation of each possible enantiomer [20]. The prepared ligands were subjected to docking studies against the protein.

#### 3.2.5. Molecular Docking Study

The molecular docking study of the prepared ligands with the protein was performed using the glide docking protocol [21]. Glide docking was performed with Extra Precession (XP) as the most precise docking mode for a small number of ligands. The XP mode assisted in identifying the potential lead, based on the most favorable affinity with the binding sites via the anchor-and-grow methodology. Thus, it allowed an excellent resolution. For each compound, the top-score docking poses were chosen for the final ligand target interaction analysis, which employed the XP interaction visualizer of Maestro 11.1 software. The validation of the docking procedure was first evaluated by the re-docking of the co-crystalized ligands into the tubulin-binding sites.

#### 3.2.6. Binding Energy Calculation

For the MMGBSA calculations, the prime MMGBSA module of Schrodinger Maestro was used. Herein, the pose viewer file was used as obtained from the molecular docking simulation study. VSGB (as a solvent model) and OPLS_2005 (as a force field) were used. Finally, using 0.5 as the distance of the ligand–protein flexibility, the MMGBSA-binding energy was calculated [22].

#### 3.2.7. Molecular Dynamic Studies

The docking complex of **1A2** with the tubulin protein was further subjected to molecular dynamics simulation studies to evaluate its binding stability at the active site of the protein. The dynamic studies were performed using Desmond as per the standard protocol [23]. Briefly, the docked ligand–protein complex obtained from the above-performed docking studies was solvated in TIP3P water. The solvated complex was neutralized using a concentration of 0.15 M of Na^+^ and Cl^−^ ions. The water layer thickness was set to 10 Å and the resulting complex was minimized before the simulation experiment. The prepared system was subjected to MD studies for 100 ns at 300 K and 1.01325 bar pressure. A root mean square deviation (RMSD) was used to determine the changes in the set of atoms of a given frame concerning the reference frame. It revealed how the structural conformation changed over time, with the protein RMSD plot on the left side of the y-axis. The RMSF was used to calculate the changes in the protein residues during the simulation. The generated graph showed that the alpha helical and beta strands were rigid compared with the other loop regions, with a significant fluctuation in the N- and C-terminals of the protein sections. The protein–ligand diagram gave insights into the amino acid involved and the types of bonding interaction. After the completion, the data were extracted; these are presented in the Results and Discussion section [24].

#### 3.2.8. Tubulin Inhibitory Potential

As a standard protocol, the in vitro tubulin-binding affinity of 1A2 was evaluated against bovine tubulin via a tubulin polymerization inhibitory assay [25]. In brief, compound **1A2** or colchicine was added to 96-well plates in a 10 µM concentration (final concentration), followed by the addition of bovine tubulin (1.8 mg/mL; Sigma Aldrich, Mumbai, Maharashtra, India) suspended in an ice-cold polymerization buffer (which was earlier centrifuged for 5 min at 4 °C and the supernatant was used). After addition of the tubulin, the 96-well plate was quickly transferred to a spectrophotometer set at a temperature of 37 °C. For 2.5 h, the absorbance was measured at the wavelength of 340 nm every 10 min. The experiments were performed in triplicate and the mean of these results was expressed.

#### 3.2.9. Cell Culture and MTT-Based Assay

**Figure 7:**
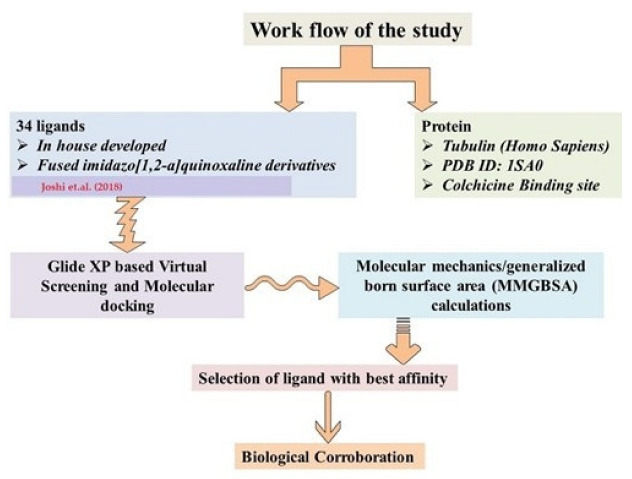
The flowchart illustration portrays the brief methodology of the proposed work [12].

Compound **1A2** was further evaluated for cytotoxicity against four cancer cell lines—MCF-7, MDA-MB-231, HCT-116, and A549—using the MTT assay [26]. In general, the cells in a concentration of 1 × 10^4^ cells/well were seeded of the respective cell line and left overnight in the incubator for adherence to the surface of the plate. After the adherence, the target compound or colchicine was added to the respective wells (*n* = 3) with varied different concentrations and incubated in a CO_2_ incubator for 48 h. For the control experiment, drug-free media were used for the comparison. After 48 h of treatment, the remaining compounds were removed and the cells were washed with PBS (phosphate buffer saline), followed by the addition of 20 μL of an MTT dye solution (5 mg/mL). The plate was again incubated for 4 h in the dark at room temperature. After the completion of the incubation period, the remaining MTT solution was removed and the formed formazan crystals were dissolved using 100 μL dimethyl sulfoxide (DMSO). The plate was shaken on a plate shaker and the absorbance was recorded at 570 nm using a microplate reader. The results were expressed as the IC_50_ (half of the maximal inhibitory concentration) values of the compounds against each cell line. Figure 7 illustrates a summary of the methodology adopted.

## 4. Conclusions

The initial in vitro activity is the critical indicator for shifting a drug candidate to the next phase during drug development. Nevertheless, most synthesized drug molecules cannot exhibit an optimum activity in the initial in vitro phase and there is a maximum probability of failure during drug development. These synthesized molecules can also be considered as a lead, and further modifications to them are worthwhile. Thus, starting with an already synthesized simplest form of a lead molecule and building other groups onto it to produce several primary scaffolds and filtering them, based on the in vitro activity, is one of the systematic approaches that can be implemented in anticancer drug discoveries. In the current work, we explored the identification of potential tubulin inhibitors with anticancer assets from a library of 34 fused imidazo[1,2-*a*]quinoxaline derivatives using computational tools, including virtual screening, molecular docking, and molecular mechanics. The molecules were rationally chosen considering the pharmacophoric features of colchicine and EAPB203—approved and investigational drugs, respectively—known to inhibit tubulin and possessing potential anticancer assets. The analysis revealed that 1A2 possessed the best affinity and binding energy within the CBS. This was further corroborated using an in vitro assay on tubulin enzymes, and the anticancer potential was established using an MTT assay. Thus, we successfully applied the utility of in silico techniques to identify the best plausible leads from fused azaheterocycles. The compound with the best affinity was further validated using in vitro-based biological methods, and its tubulin inhibitory potential and, finally, its anticancer assets were established.

## Figures and Tables

**Table 1:** Compilation of binding affinity scores (glide scores) and parameters of molecular mechanics.

Sr. No.	Compound Code	G-Score (kcal/mol)	MMGBSA_dG_Bind	MMGBSA_dG_Bind_Coulomb	MMGBSA_dG_Bind_Covalent	MMGBSA_dG_Bind_Hbond	MMGBSA_dG_Bind_Lipo	MMGBSA_dG_Bind_vdW
1.	**1A1**	−8.5	−40.6	−1.7	1.6	−0.5	−11.9	−43.8
2.	**1A2**	−11.5	−55.0	−7.6	1.9	−1.3	−17.3	−52.3
3.	**1B2**	−9.1	−48.5	−12.6	9.1	−0.1	−18.7	−51.1
4.	**1B3**	−7.5	−42.5	−8.6	−0.9	−0.6	−15.4	−35.5
5.	**1B4**	−8.5	−41.3	−19.3	1.6	−1.2	−11.1	−38.7
6.	**1B5**	−8.5	−38.8	−12.8	0.5	−2.0	−11.5	−37.9
7.	**1B6**	−7.9	−31.8	−6.7	−8.8	−2.3	−17.4	−42.3
8.	**1C2**	−9.3	−50.3	−11.2	1.8	−0.6	−18.0	−45.9
9.	**1C3**	−8.4	−37.8	−11.3	3.3	−1.1	−15.2	−39.8
10.	**1C4**	−8.0	−37.5	−4.1	1.3	−1.5	−15.2	−38.8
11.	**1C5**	−8.3	−37.2	−3.8	0.1	−1.0	−14.1	−35.0
12.	**1C6**	−7.3	−37.0	−9.9	0.4	−0.6	−9.9	−28.6
13.	**1C7**	−9.3	−50.2	−0.4	0.8	−0.7	−19.8	−46.6
14.	**1C8**	−9.1	−39.6	−8.9	8.1	−1.2	−18.4	−50.4
15.	**1C9**	−8.9	−35.7	−17.0	2.8	−2.4	−8.8	−34.9
16.	**1C10**	−8.3	−33.7	−7.8	3.0	−1.0	−14.3	−33.8
17.	**1C11**	−8.2	−33.2	−2.1	0.7	−0.6	−10.0	−35.6
18.	**1D1**	−8.2	−38.9	−6.2	−0.7	−21.6	−31.4	−38.9
19.	**1D2**	−9.2	−42.3	−13.9	0.9	−1.3	−12.4	−38.5
20.	**1E1**	−8.1	−31.7	−7.8	0.6	−0.8	−11.5	−30.4
21.	**1E2**	−8.1	−34.9	−2.1	1.8	−1.7	−11.2	−38.9
22.	**1E3**	−9.5	−26.4	4.0	5.8	−0.6	−18.9	−47.2
23.	**1E4**	−8.1	−28.4	−0.6	2.4	−0.6	−14.8	−36.9
24.	**1E5**	−8.1	−31.5	−5.5	1.5	−1.0	−11.2	−37.7
25.	**1E6**	−8.0	−32.2	−7.6	2.6	−1.0	−13.8	−37.1
26.	**1E7**	−9.4	−31.4	−3.5	3.6	−0.8	−15.3	−40.6
27.	**1E8**	−9.5	−52.5	−27.8	9.5	−1.5	−23.7	−52.9
28.	**1E9**	−7.9	−28.6	−8.9	3.0	−1.3	−11.7	−34.1
29.	**1E11**	−7.1	−35.8	−7.0	1.4	−0.9	−14.5	−32.1
30.	**1E12**	−9.4	−41.7	−11.7	8.7	−0.5	−21.8	−55.0
31.	**1E13**	−9.2	−41.6	−11.7	7.5	−0.3	−19.1	−51.5
32.	**1E14**	−8.1	−38.5	−7.6	5.7	−0.1	−18.2	−49.6
33.	**1E15**	−8.5	−38.1	−11.7	3.1	−1.2	−15.0	−39.4
34.	**1E16**	−7.8	−42.7	−11.4	1.8	−1.3	−19.3	−37.9
35.	**Colchicine**	−9.2	−51.7	−11.0	3.5	−1.1	−17.7	−46.7

**Table 2:** In vitro antiproliferative activity (IC_50_ in µM) of compound **1A2** against MCF-7, MD-MB-231, HCT-116, and A549 cancer cell lines.

Compound	IC_50_ ± SEM (µM)			
	MCF-7	MDA-MB-231	A549	HCT-116
1A2	4.33 ± 0.31	6.11 ± 0.23	5.87 ± 0.31	5.44 ± 0.18
Colchicine	5.11 ± 0.33	5.14 ± 0.35	6.55 ± 0.41	5.54 ± 0.33

## Data Availability

Data is unavailable due to privacy or ethical restrictions.
